# Does Open Access Improve the Process and Outcome of Podiatric Care?

**DOI:** 10.4021/jocmr545w

**Published:** 2011-05-19

**Authors:** James S. Wrobel, Michael L. Davies, Jeffrey M. Robbins

**Affiliations:** a Captain James A. Lovell Federal Health Care Center, North Chicago, IL, and Scholls Center for Lower Extremity Ambulatory Research (CLEAR) at Rosalind Franklin University of Medicine, North Chicago, IL, USA; b Systems Redesign Veterans Affairs Central Office, USA; c Podiatric Services, Veterans Affairs Central Office and Cleveland VA Medical Center, Department of Veterans Affairs, Cleveland, OH, USA

## Abstract

**Background:**

Open access to clinics is a management strategy to improve healthcare delivery. Providers are sometimes hesitant to adopt open access because of fear of increased visits for potentially trivial complaints. We hypothesized open access clinics would result in decreased wait times, increased number of podiatry visits, fewer “no shows”, higher rates of acute care visits, and lower minor amputation rates over control clinics without open access.

**Methods:**

This study was a national retrospective case-control study of VHA (Veterans Hospital Administration) podiatry clinics in 2008. Eight case facilities reported to have open podiatry clinic access for at least one year were identified from an email survey. Sixteen control facilities with similar structural features (e.g., full time podiatrists, health tech, residency program, reconstructive foot surgery, vascular, and orthopedic surgery) were identified in the same geographic region as the case facilities.

**Results:**

Twenty-two percent of facilities responded to the survey. Fifty-four percent reported open access and 46% did not. There were no differences in facility or podiatry panel size, podiatry visits, or visit frequency between the cases and controls. Podiatry visits trended higher for control facilities but didn’t reach statistical significance. Case facilities had more new consults seen within 30 days (96%, 89%; P = 0.050) and lower minor amputation rates (0.62/1,000, 1.0/1,000; P = 0.041).

**Conclusions:**

The VHA is the world’s largest managed care organization and it relies on clinical efficiencies as one mechanism to improve the quality of care. Open access clinics had more timely access for new patients and lower rates of minor amputations.

**Keywords:**

Health care; Quality; Access; Evaluation; Delivery of health care; Amputation; Amputation prevention

## Introduction

“Open” access to clinics has been put forth as a mechanism to improve access to healthcare. Open access intends to offer appointments at the patients convenience by eliminating waiting times for appointments. However, providers are sometimes resistant to adopting open access because of a fear of being inundated with visits for trivial complaints, thus sacrificing access for true clinical urgencies.

Several studies have supported the use of open access scheduling for primary care practices [1-6]. These process improvement strategies have taken place in a variety of clinical settings including managed care, military and Veterans Health Administration (VA) facilities. Reductions in wait times range from 13 - 78 days in the VA setting [7, 8].

There are challenges to implementing and sustaining this strategy [9, 10]. There has been a report suggesting seasonality of demand may decrease predictability [9]. Other barriers consist of practice styles, panel compositions, patient preferences, and time varying demand patterns [10].

While these initial reports are encouraging for primary care practices, it is unclear whether this model would be appropriate for sub-specialty practice that may have a higher and more unpredictable demand for time-consuming procedures. One report investigated the appropriateness of direct scheduling for endoscopy based type of practitioner [11]. In a review of 310 consecutive direct referrals from surgeons and primary care for esophagealgastroduodenoscopy (EGD) and colonoscopy, Mahajan and colleagues reported 97% v. 81% appropriate referrals for EGD with primary care physicians and surgeons respectively. For colonoscopies, the numbers were 85% and 67% [11]. In the VA setting, urology clinics improvements in wait times ranged from 20 - 25 days better than the average wait time [7, 8]. While open access has demonstrated improvement in wait times, patient and provider satisfaction, it is unclear what the consequences may be on other measures of efficiency and quality, such as, no shows, visits, case mix, process measures, and outcomes. Based on the above, we hypothesized that open access clinics would result in decreased wait times, increased number of podiatry visits, fewer no shows to podiatry clinics, have higher rates of acute care diagnostic and procedure codes (i.e., paronychia, abscess, cellulitis, ulcer, arthrocentesis, matricectomy, tendon sheath injection, etc.), and lower minor amputation rates over control clinics that did not institute open access.

## Materials and Methods

### Experimental design

The study was retrospective case: control design of VA podiatry clinics examining data from fiscal year 2008. Case facilities were defined from email survey of stations reporting “open” access to podiatry clinics at least one year prior to the start of the study. Open access was defined as providing patient access through the provision of a walk-in clinic, setting aside varying number of appointments/day that could not be filled > 48 hours in advance, or have a set process for allowing walk-in patients. For each case facility, two control facilities were selected. These were selected by the Director of Podiatric Services (JMR) due to his familiarity with the structural characteristics of the hospitals. Control facilities were defined as centers in the same geographic regional quadrant of the country. When possible these were matched within the same Veteran Integrated System Network (VISN). Control facilities had similar structural features (i.e., podiatry full-time equivalent employees (FTEE), health tech (nail care tech), podiatric medicine and surgery residency program, reconstructive foot surgery program, vascular, and orthopedic surgery access). The study was approved by the Institutional Review Board.

### Survey

In May 2009, a three question short answer survey was sent via email to all podiatrists in the VA system by the Director of Podiatric Services (JMR). Of the 172 facilities, 37 facilities responded to the survey reflecting a 22% response rate. Of the responders, twenty facilities (54%) reported open access clinics and 17 (46%) did not.

### Data sources

Ethical approval for the conduct of the study was received from the VA research and development committee and institutional review board of the lead author’s institution. Data sources for each facility came from specific queries made to the ProClarity Cube. The VA annual clinical productivity report was also used to define facility wait times, no-shows, and top 10 ICD-9 codes for each visit as sampled in the month of March 2008.

### Main outcome measures

The main outcome measure was the wait times as defined as % of new consults meeting the 30 day appointment timeliness goals. Secondary measures included the number of podiatry visits, no show visits, acute care signature, and outcomes of yearly age-standardized amputation rate. The acute care signature was determined from rates of acute care ICD-9 diagnostic or CPT treatment codes per 100 podiatry visits. These included visits for paronychia (infected ingrown toenail), cellulitis (soft tissue infection of the foot or ankle), lower extremity skin ulcer, injection of the foot or ankle (e.g., arthrocentesis or tendon sheath injection), removal of a toenail under local anesthesia, or attempted permanent removal of a toenail (e.g., matricectomy).

### Facility masking

The study was not entirely blinded. The data was retrospective. Author JMR aggregated data at the facility-level. JSW was responsible for the final analysis.

### Statistical analyses and sample size calculation

All hypothesis testing was calculated using a two-tailed two-sample t test with unequal variances assumption using Welchs formula. Inter-facility comparisons were made using one-way ANOVA when there were equal variances as determined by Bartletts test. Statistical significance was set at 0.05. Based on our pilot data from March 2006 and 2007, a sample of one center providing 12.5% of new consults an appointment within 30 days while another center provided 94.1% of new consults having an appointment within 30 days. Based on this assumption, 8 case facilities were required with an alpha of 0.05 and power of 90%. This sample size provided ~ 99% power to detect a 10% increase in yearly visits and 69% power to detect a 5% increase in visits. It also provided 85% power to detect a decrease in “no-show” visits from 15% down to 2%. A change in national amputation rates was not expected [12]. Despite this, there is 100% power to observe another 9.5% yearly reduction in total amputation count and 100% power to detect a 10% increase in ulcer debridement and corn or callus debridement rates. For amputation hypotheses, pilot data from one VISN revealed a decrease in total amputation count from FY’05 (233) to FY’06 (211). As age standardized rates of amputation have declined for many years in the VA, the interpretation of this section of the analysis has a number of caveats. All calculations were made using STATA 10.1 (College Station, TX, USA).

## Results

**Table 1:** Descriptive Characteristics of Case and Control Centers for Fiscal Year 2008

Facility Features	Open Access	No Open Access	P value
	(N = 8)	(N = 16)	
Unique Patients			
Facility	45,131	46,528	0.98
Podiatry	2,068	2,767	0.12
Podiatry Encounters	4,691	6,911	0.08
Visit Frequency	2.21	2.44	0.12
% With Residency Program	50%	44%	1.00
% Active Surgery	50%	38%	0.67
Process Measures			
% meeting 30 day wait time	96%	89%	0.05
% no show visits	8.6%	9%	0.25
Acute Care Signature (rates per 100 podiatry visits)			
Paronychia	3.7	2.5	0.32
Nail avulsion	2.0	1.6	0.65
Foot & ankle injections	8.6	9.1	0.90
Nail matricectomy	1.2	1.7	0.44
Cellulitis	2.6	2.0	0.38
Lower extremity ulcer	19	20	0.84
Outcome Measures			
Major amputation rates (per 1,000)	0.34	0.59	0.09
Minor amputation rates (per 1,000)	0.62	1.0	0.04
Hi-Lo ratio	0.51	0.60	0.33

The descriptive characteristics of the facilities are described in Table 1. There were no differences in the sizes of the facilities, podiatry panel size, number of podiatry visits, or visit frequency between the case and control facilities. Podiatry panel sizes and visits trended higher for the control facilities; however, they did not reach statistical significance. Interestingly, the visit frequency and no-show rates trended lower for case facilities, but were also not significant. There were no differences in the acute care signature (Table 1). Case facilities had higher percentages of new consults being seen within 30 days (96% v. 89%, P = 0.050). They also had lower minor amputation rates (0.62 v. 1.0, P = 0.041). After adjusting for the differences in visits and minor amputation rates, the effect size for podiatry consults in 30 days did not change.

## Discussion

Our main finding was case facilities adopting open access strategies had statistically significant more new consult patients being seen within 30 days (96% v. 89%; P = 0.050). It should be noted that these rates were already high. While some providers may be hesitant to institute open access because of a fear of increased demand, this was not borne out in our data as the visit frequency was actually lower for open access clinics (2.21 v. 2.44; P = 0.12) although not statistically significant. The authors were surprised by the finding that open access facilities demonstrated 58% lower major (P = 0.09) and 62% lower minor amputation rates (P = 0.04). This did not appear to be the result of higher rates of acute care practices as there were no differences in treatment rates for lower extremity ulcer care, lower extremity cellulitis, infected ingrown toenails, foot & ankle injections, or temporary or permanent toenail removal. This finding suggests amputation rate differences may be related to improved access rather than differences in practice structure or acute care behavior.

Many quality improvement experts recommend improving the process of high risk foot care through use of stratified foot risk exams [13]. Using this approach [14] in another managed care setting, Lavery and colleagues described a 47% reduction in amputations, 38% reduction in hospital admissions, and 70% reduction in skilled nursing facility admissions observed over a 24 month period [15]. This sample of centers from the VA system demonstrates a weighted average of 0.57 for the Hi-Lo ratio. This ratio is the high or major amputation rate (e.g., below knee and above knee) divided by the low or minor amputation rate (e.g., toes or partial foot). The VA Hi-Lo ratio of 0.57 compares favorable with the US age-adjusted estimates ranging from 0.7 - 0.8 [16].

It is also interesting that while the centers were almost equal in size in terms of total unique patients, the podiatry panels trended towards larger panels and increased visits at the control facilities. This could represent that risk-based care results in better outcomes.

There are potential limitations to this study. Selection bias could have been present due to the low response rate in general or a difference in perception of institution of open access. Open access facilities may represent centers with better coordination of care, policy and improvement measurement, or Microsystems of foot care [17-19]. This study was retrospective and relied on ICD-9 coded data from a national database although the data was not subject to recall bias as ICD-9 data was coded on the day of the visit. Data were not aggregated prospectively at the facility-level.

### Conclusion

The VA is the worlds largest managed care organization. This quasi-capitated and quasi-closed system relies on clinical efficiencies as one mechanism for improving the quality of care. Open access may be a mechanism to improve access to care for higher risk patients that stand to benefit most from this care. We found open access clinics had better rates of new consult patients being seen within 30 days and lower rates of minor amputations.
